# Immunotherapy in cervical cancer: an innovative approach for better treatment outcomes

**DOI:** 10.37349/etat.2025.1002296

**Published:** 2025-03-02

**Authors:** Treshita Dey, Sushma Agrawal

**Affiliations:** Alma Mater Studiorum Università di Bologna, Italy; Department of Radiotherapy, Sanjay Gandhi Post Graduate Institute of Medical Sciences, Lucknow 226014, India

**Keywords:** Immunotherapy, cervical cancer, radio-immunotherapy, metastatic cervical cancer, immune-check point inhibitors

## Abstract

Cervical cancer remains a significant global health challenge, ranking as the fourth most common cancer among women. Persistent infection with high-risk human papillomavirus (HPV) is the primary etiological factor, leading to immune evasion mechanisms that promote tumor development and progression. Immunotherapy has emerged as a transformative approach in the management of cervical cancer, aiming to restore and enhance the body’s immune response against tumor cells. Checkpoint inhibitors targeting programmed death-1 (PD-1) and its ligand (PD-L1) have shown promising results in patients with advanced or recurrent cervical cancer. Pembrolizumab, a PD-1 inhibitor, has been approved for PD-L1-positive cervical cancer, demonstrating durable responses. However, low response rates necessitate exploration of combination strategies. Trials are underway combining checkpoint inhibitors with chemotherapy, radiation, or other immunotherapeutic agents to enhance efficacy. Therapeutic vaccines targeting HPV antigens, such as E6 and E7 oncoproteins, are also a focus of active research. These vaccines aim to elicit robust cytotoxic T-cell responses, offering a potential strategy for early intervention and disease control. Adoptive T-cell therapies, including engineered T-cell receptor (TCR) and chimeric antigen receptor (CAR)-T cells, represent cutting-edge advancements, though challenges with tumor heterogeneity and off-target effects persist. However, challenges such as limited response rates and immune evasion mechanisms remain. The tumor microenvironment (TME) in cervical cancer, characterized by immunosuppressive cells and cytokines, poses a significant barrier to effective immunotherapy. Emerging approaches targeting the TME, such as cytokine modulation, hold promise in overcoming resistance mechanisms. Key gaps include a lack of biomarkers for patient selection, insufficient understanding of TME dynamics, and suboptimal strategies for overcoming antigen heterogeneity and immune resistance. This review addresses these issues by providing a comprehensive analysis of the current landscape of cervical cancer immunotherapy, identifying critical barriers, and highlighting emerging approaches, such as combination therapies, novel immune targets, and strategies to modulate the TME, to guide future research and clinical practice.

## Introduction

Cervical cancer is one of the most common malignancies affecting women worldwide, with significant morbidity and mortality, particularly in low- and middle-income countries (LMIC). As per the GLOBOCAN 2020, cervical cancer is the most common gynaecological cancer and is ranked 8th most common cancer with an annual incidence of 0.6 million [1]. Persistent infection by high-risk types of human papillomavirus (HPV), primarily HPV16 and HPV18 has been linked to the pathogenesis of cervical cancer [2]. Although widespread HPV vaccination programs have dramatically reduced the incidence of cervical cancer in most developed countries with high vaccine coverage, it still remains a significant global problem in LMIC.

Traditionally, the management of cervical cancer has relied on a combination of surgery, radiation therapy, and chemotherapy, depending on the stage of the disease. While these conventional therapies can be effective, particularly in early-stage cancers, they often fail in advanced or recurrent cases. The prognosis for metastatic or recurrent cervical cancer remains poor, highlighting the need for new therapeutic strategies. With conventional chemotherapy, the response rate is around 30% with a median survival of 7 months [3].

In recent years, immunotherapy has emerged as a promising approach in the treatment of various cancers, including cervical cancer. Immunotherapy leverages the body’s immune system to recognize and eliminate tumour cells. Immune checkpoint inhibitors (ICI) have shown efficacy in cervical cancer by targeting immune checkpoints which tumours exploit to evade immune detection. These therapies have demonstrated durable responses in other malignancies in patients with advanced or recurrent cervical cancer, where few options previously existed and even have been recommended in first line settings.

This review will explore the role of immunotherapy in the treatment of cervical cancer, examining current evidence, mechanisms of action, and future directions. As the field evolves, immunotherapy could potentially transform the landscape of cervical cancer treatment, offering hope to patients with limited treatment options.

## Background: cervical cancer and immunotherapy

**Figure 1:**
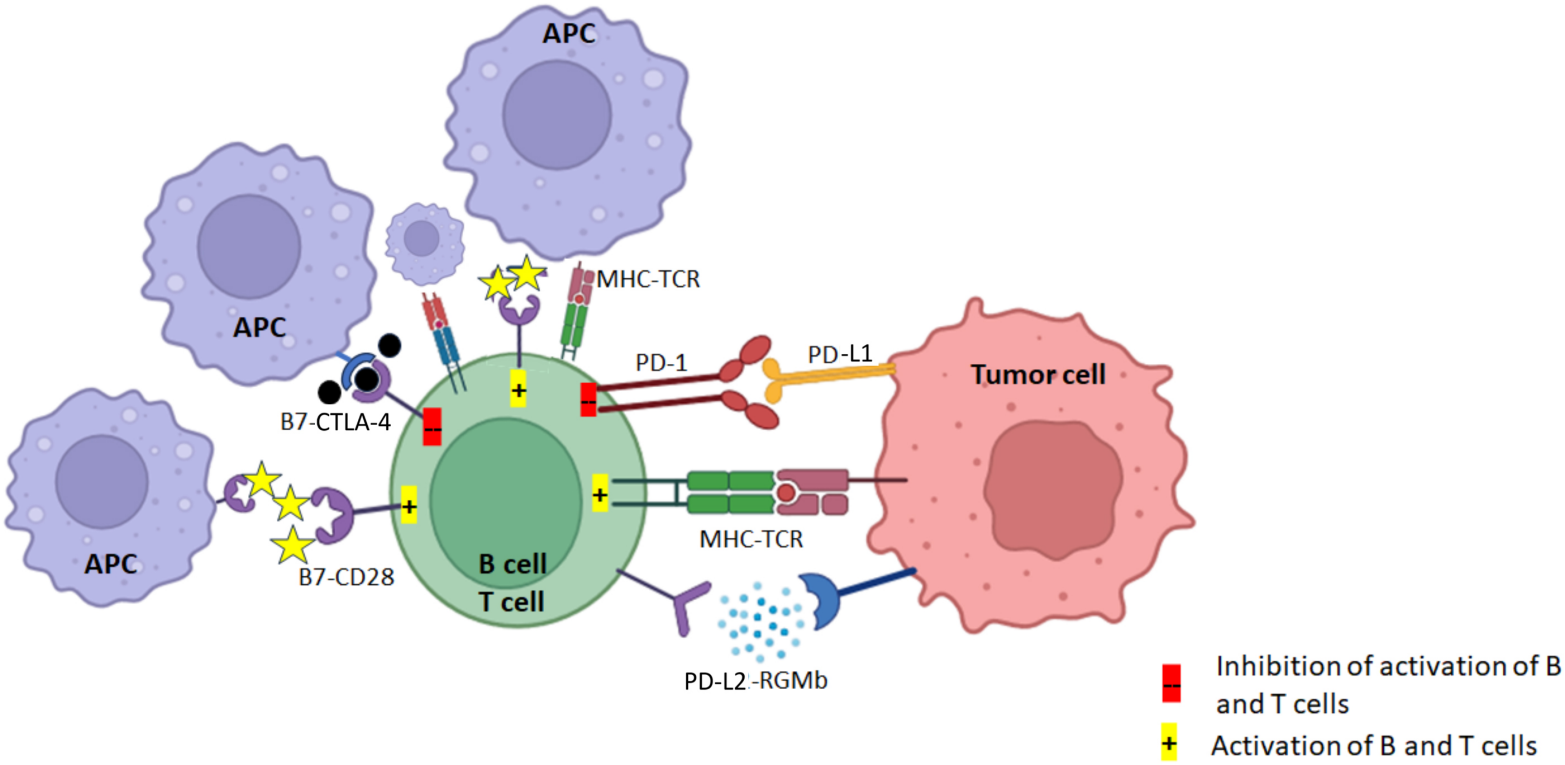
**PD-1/PD-L1 and CTLA-4 pathways: the TCR of CD8^+^ T cell activates upon recognizing the tumor antigen presented on MHC class I and consequently induces the expression of PD-L1 on tumor cells**. PD-L1 conjugates the elevated PD-1 on T cell surface, triggering inhibitory effect of PD-1/PD-L1 axis. In addition, CD28 and ligands B7 (CD80 and CD86) are exposed to the TME, T cells become unreactive or eliminated by programmed cell death. CTLA-4 competes with CD28 to bind the costimulatory B7 receptors on antigen presenting cells. CTLA-4 transmits an inhibitory signal to T cells, whereas CD28 transmits a stimulatory signal. APC: antigen-presenting cell; CTLA-4: cytotoxic T-lymphocyte-associated protein 4; MHC: major histocompatibility complex; TCR: T-cell receptor; PD-1: programmed death-1; PD-L1: programmed death-ligand 1; TME: tumor microenvironment. Created in BioRender. Dey, T. (2025) https://BioRender.com/b08y621

**Figure 2:**
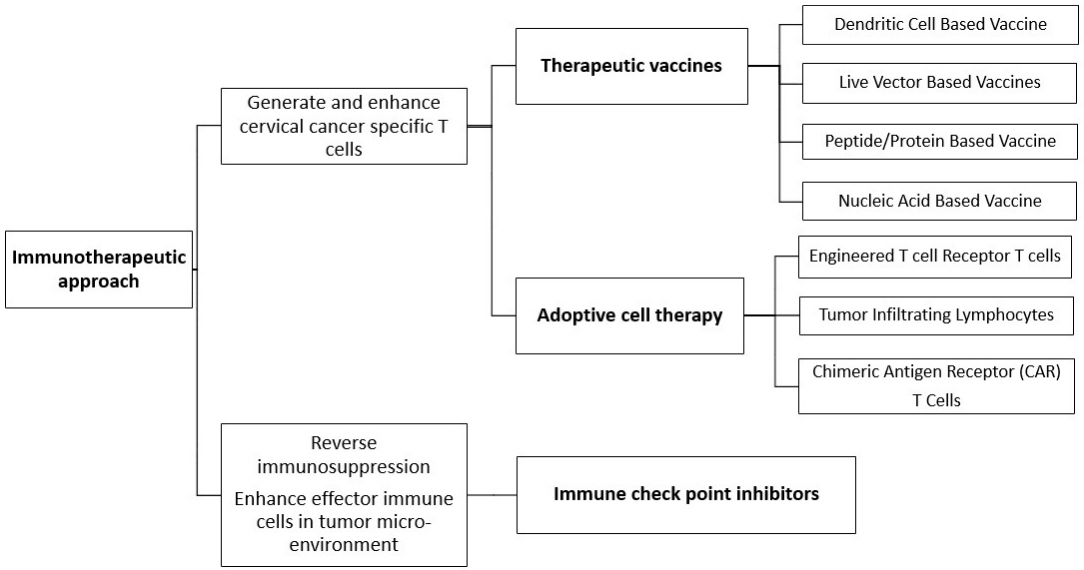
Various immunotherapeutic approaches against malignant cells, therapeutic vaccines and adoptive cell therapy generate and enhance cancer-specific T cells whereas immune checkpoint inhibitors

Cervical cancer is strongly associated with persistent infection by high-risk HPV strains, particularly HPV16 and HPV18. These viruses induce oncogenic changes by expressing E6 and E7 oncoproteins, which disrupt tumor suppressor pathways and promote immune evasion [4]. E6 binds to and promotes the degradation of the tumor suppressor *p53* gene, impairing cell cycle arrest and apoptosis. Simultaneously, E7 binds to the retinoblastoma protein (Rb), releasing E2F transcription factors, which drive uncontrolled cell cycle progression. This dual inactivation of *p53* and Rb leads to genomic instability, unchecked cell proliferation, and resistance to apoptosis, ultimately resulting in malignant transformation. Given the immunogenic nature of HPV-related antigens, cervical cancer presents a favourable target for immunotherapeutic approaches, including ICI, therapeutic vaccines, and adoptive cell therapy (Figures 1 and 2).

### Immune check point inhibitors

ICI enhance the immune system’s ability to detect and destroy cancer cells by blocking proteins that act as “checkpoints” to prevent excessive immune responses. Tumor cells often exploit these checkpoints to evade immune detection. ICI mainly target two pathways: PD-1 and cytotoxic T-lymphocyte-associated protein 4 (CTLA-4). CTLA-4 acts at the early stage of T-cell immune response primarily in lymph nodes, but PD-1/PD-L1 plays an important role at the later stage of T-cell immune response primarily in peripheral tissues [5].

#### PD-1/PD-L1 pathway

PD-1 is a receptor on activated T-cells that regulates immune responses. When it binds to its ligands, PD-L1 or PD-L2, it downregulates T-cell activity to promote immune tolerance and prevent autoimmunity [6]. Tumor cells can overexpress PD-L1 to protect themselves from immune attack by binding to PD-1, which inhibits T-cell activity. ICI targeting PD-1 (e.g., pembrolizumab, nivolumab) or PD-L1 (e.g., atezolizumab, durvalumab) block this interaction, effectively releasing the “brake” on T-cells. As a result, T-cells remain active and capable of recognizing and destroying tumor cells [7–9].

#### CTLA-4 pathway

CTLA-4 is another immune checkpoint receptor that modulates T-cell activity early in immune responses. Normally, T-cell activation requires two signals: antigen recognition via the major histocompatibility complex (MHC) and T-cell receptor (TCR) interaction, and a co-stimulatory signal via CD28 binding to B7 molecules (CD80/CD86) on antigen-presenting cells (APCs) [10]. CTLA-4 competes with CD28 for binding to B7 molecules but delivers an inhibitory signal, dampening T-cell activation [11]. CTLA-4 inhibitors (e.g., ipilimumab) block CTLA-4 from binding to B7, preventing the inhibitory signal and enhancing T-cell activation and proliferation. This results in increased immune activity against tumors.

By blocking these immune checkpoints, ICI restore the immune system’s ability to target tumors, offering a potent approach to cancer therapy. However, the broader activation of T-cells can also lead to immune-related adverse events (AE), as the immune system may attack normal tissues due to reduced tolerance.

### Role of ICI in neoadjuvant immunotherapy

Neoadjuvant immunotherapy leverages the presence of an intact tumor in situ to modulate the immune system, by providing a constant source of tumor antigens, which can stimulate a more robust immune response. Additionally, it offers the opportunity to generate systemic immune memory through the release of tumor antigens during immunotherapy-induced tumor cell death, facilitating long-term surveillance and reducing recurrence risk. Neoadjuvant ICI reshape the tumor microenvironment (TME) by reducing immune-suppressive cells, such as regulatory T cells (Tregs) and myeloid-derived suppressor cells, and promoting the infiltration of cytotoxic CD8^+^ T cells. Combining neoadjuvant immunotherapy with standard treatments, such as radiation or chemotherapy, can further enhance immunogenic cell death, creating a synergistic effect. Clinical trials exploring neoadjuvant immunotherapy in cervical cancer are limited but growing.

A phase 2 trial from China (ChiCTR2200061097) presented in ASCO 2024 evaluated the efficacy and safety of camrelizumab (PD-1 inhibitor) + taxane-platinum based neoadjuvant chemo-immunotherapy, followed by chemoradiotherapy (CRT) and camrelizumab maintenance in locally advanced cervical cancer (LACC) [12]. Among 55 evaluable patients, 98.1% patients achieved complete remission after brachytherapy. At 1-year, progression-free survival (PFS) was 90.6%. Serious AE occurred in 12.7%, mostly infections. No treatment-related deaths occurred, and no new safety concerns were observed.

The multicentre NACI (NCT04516616) trial also evaluated neoadjuvant chemo-immunotherapy using camrelizumab in LACC followed by surgery or CRT [13]. The preliminary results showed 19% of patients achieving complete response and 79% partial response. Grade 3-4 treatment-related AE were manageable, no serious AE or treatment-related deaths were observed.

### Role of ICI in concurrent chemoradiation

#### Rationale

Radiotherapy (RT) exerts its effects not only through direct tumor cell killing by inducing DNA damage but also via significant immunomodulatory actions. The radiation-induced tumor cell death releases tumor antigens, promoting an immune response by increasing antigen presentation through dendritic cells (DCs) leading to the activation of cytotoxic T-cells (CTLs). RT also induces immunogenic cell death, characterized by the release of damage-associated molecular patterns (DAMPs), such as calreticulin and high mobility group protein B1 (HMGB1) [14, 15]. These molecules enhance the recruitment and activation of immune cells in the TME, shifting it from immunosuppressive to immune-reactive state. RT can stimulate release of pro-inflammatory cytokines (e.g., interferon-gamma) and chemokines (e.g., CXCL9, CXCL10), which can recruit more immune cells, especially CTLs, to the tumor site [16]. RT can also increase the MHC expression on the surface of cancer cells, enhancing antigen presentation [17].

TME is typically immunosuppressive, characterized by factors such as Tregs, myeloid-derived suppressor cells, and an abundance of inhibitory molecules like PD-L1 [18]. RT can promote the upregulation of immune checkpoints like PD-L1 on tumor cells, which, while providing a potential mechanism of immune escape, also presents a target for blockade by PD-1/PD-L1 inhibitors [19]. When combined with RT, ICI can amplify the immune response by ensuring that the activated T-cells, which are primed by the tumor antigens released during RT, are not suppressed by inhibitory pathways [20]. This dual action-enhancing T-cell activation can lead to a more robust antitumor response.

Another critical mechanism is the “abscopal effect”, where local RT leads to systemic antitumor responses, including regression of non-irradiated metastatic tumors [21]. This phenomenon is thought to be mediated by the immune system, where RT activates a tumor-specific immune response that not only targets the primary tumor but also metastases. While the abscopal effect is rare when RT is used alone, its occurrence is more frequent when combined with ICIs, as these agents prevent the suppression of systemic immune responses [22].

Furthermore, RT-ICI combination addresses tumor heterogeneity [23]. While RT is effective at targeting the localized tumor, ICI can target distant micro-metastatic sites by reinforcing systemic immune surveillance. The integration of RT with ICI also allows for optimization of dosing and sequencing strategies. For instance, low-dose RT may preferentially modulate immune environment without causing excessive tissue damage, while high-dose RT can act as potent immune adjuvant when administered concurrently with ICI [24, 25]. This synergy creates a more favorable TME, enhancing immune infiltration and reducing resistance.

#### Clinical evidence

The KEYNOTE-A18 trial was a phase 3, randomized, double-blind study evaluating the combination of pembrolizumab with CRT in patients with FIGO stage IB2–IIB with node-positive disease or stage III–IVA [26]. The results were significant, showing a 30% reduction in the risk of progression or death with pembrolizumab. Grade ≥ 3 AE occurred in 75% of patients in the pembrolizumab group, slightly higher than in the placebo group. The Food and Drug Administration (FDA) approved pembrolizumab for this indication in January 2024 based on these results [27].

The CALLA trial evaluated the addition of durvalumab, to standard CRT in LACC [28] on 770 patients. Unfortunately, the trial did not meet its primary endpoint, since durvalumab did not significantly improve PFS compared to concurrent chemoradiation (CCRT) alone. However, some benefit was noted in patients with higher PD-L1 expression. AE were similar in both groups, with manageable side effects, though slightly higher immune-related events were seen in the durvalumab arm. There are some other trials, mostly phase 1 with smaller sample size which have also looked at ICI concurrently with RT that have been summarised in Table 1 [29–31].

**Table 1:** Summary of studies highlighting the role of immunotherapy in radical treatment in cervical cancer

**Role**	**Author, year**	**Type**	**Sample size**	**Inclusion criteria**	**Treatment**	**Median follow up**	**Disease response**	**Toxicity**
Neo-adjuvant	Chen et al. [12], 2024	Single-arm, phase 2 trial	55	FIGO 2018 stage IIB–IVA disease), ECOG PS score 0–2	2 cycles neoadjuvant camrelizumab + TP, CCRT with concurrent triweekly camrelizumab + cisplatin, maintenance camrelizumab upto 1 year	9.5 months	98.1% complete remission 5.5% treatment failure 1-year PFS 90.6%	Grade 3–4 neutropenia (18.2%), thrombocytopenia (9.1%) and anemia (27.3%). Serious AE: 12.7%, most commonly due to infectious complications
	Li et al. [13], 2024	Multicentre single-arm, phase 2 trial	85	FIGO 2018 IB3, IIA2, or IIB/IIIC1r, ECOG PS 0–1	1 cycle TP + 2 cycles TP-camrelizumab, CCRT for stable disease or progressive disease, SURGERY for complete or partial response	11 months	19% complete response, 79% partial response	Grade 3–4 lymphopenia 25%, neutropenia 12%, leukopenia 8%
Concurrent	Lorusso et al. [26], 2024	Randomised phase 3	1,060	High-risk, LACC	5 cycles of pembrolizumab (200 mg) or placebo q3 weeks plus CCRT, & 15 cycles of pembrolizumab (400 mg) or placebo q6 weeks	17.9 months	2-year PFS 68% in pembrolizumab-CCRT group vs. 57% in placebo-CCRT HR for progression 0.7 2-year OS: 87% in pembrolizumab-CCRT and 81% in the placebo-CCRT HR for death 0.73	Grade ≥ 3 AE 75% in pembrolizumab-CCRT and 69% in placebo-CCRT
	Rodrigues et al. [29], 2024	Multicentre phase 1	16	Stage IB3–IVA cervical cancers	Concurrent nivolumab + CCRT (with cisplatin) and maintenance (till 6 month post CCRT) (13 cycles)	16.6 months	1-year PFS 81.2%	3/16 patients experienced dose limiting toxicity
	Duska et al. [31], 2020	Randomized phase 2	52	LACC (stage IB–IVA FIGO 2009)	Pembrolizumab post CRT (arm 1) or during CRT (arm 2)	4.8 months	NA	Grade ≥ 2 AE in 88%
	Mayadev et al. [30], 2020	Phase 1	32	FIGO stage IB2–IVA cervical cancer with positive pelvic, para-aortic LNs, or both	CCRT (6 weekly doses of cisplatin) + sequential ipilimumab q21 days for 4 cycles	14.8 months	1-year OS 90%, and PFS 81%	9.5% self-limiting grade 3 AE (lipase increase; dermatitis)
	Chen et al. [32], 2025	Single-center, single-arm, prospective phase 2	82	Stage IB3–IVA (2018 FIGO)	CCRT with cisplatin and toripalimab and adjuvant 6 cycles of cisplatin-paclitaxel-toripalimab	21 months	1 and 2-year PFS: 90.9%, 88.5% 1 and 2-year OS: 98.6%, 95.2%	Grade ≥ 3 AE: 20.7%
	Monk et al. [28], 2023	Randomised, double-blind, phase 3 trial	770	FIGO 2009 stage IB2–IIB lymph node positive, stage ≥ III any lymph node status) and ECOG PS 0–1	Durvalumab or placebo with and following CCRT, for up to 24 cycles	18.5 months	12-month PFS 76% with durvalumab and 73.3% with placebo	Grade 3–4 anaemia 20% in durvalumab vs. 15% in placebo, decreased white blood cells 10% vs. 13% serious AE 28% in durvalumab vs. 23% in placebo Five treatment-related deaths in the durvalumab

ECOG PS: Eastern Cooperative Oncology Group performance status; TP: taxane-platinum; CCRT: concurrent chemoradiation; CRT: chemoradiotherapy; PFS: progression-free survival; AE: adverse events; HR: hazard ratio; LACC: locally advanced cervical cancer

A Chinese study assessed the safety and efficacy of toripalimab (humanized IgG4k monoclonal antibody (mAb) designed to target PD-1, preventing binding with PD-L1 and PD-L2) concurrently with CCRT on 82 LACC patients [32]. There was an objective response and disease control rate of 87.8%. Median PFS and OS were not reached, with trends toward improved PFS in patients with higher PD-L1 scores and low tumor mutation burden. AE were manageable, with 20.7% experiencing grade 3 or higher events.

### Immunotherapy in metastatic or recurrent cervical cancer

Patients with metastatic or recurrent cervical cancer have limited treatment options, and their prognosis is generally poor. Historically, systemic chemotherapy has been the standard of care, but response rates are suboptimal, and outcomes are often disappointing. Cervical cancer, being driven by HPV infection, expresses viral antigens that can be recognized by the immune system, making it suitable target for immunotherapy. Additionally, TME in cervical cancer often exhibits high PD-L1 expression, which can be targeted by ICI. ICI offers a novel approach with the potential to improve survival outcomes.

The KEYNOTE-028 trial evaluated pembrolizumab in 24 patients with PD-L1-positive locally advanced, or metastatic cervical cancer that had progressed after prior treatment [33]. It showed a partial response in 17% and stable disease in 13%, with a median duration of response of 5.4 months. Median PFS was 2 months, and median OS was 11 months. In the KEYNOTE-158 trial [34], pembrolizumab showed 14.3% objective response rate (ORR) in patients with recurrent or metastatic cervical cancer. Median PFS was 2.1 months, and median OS reached 9.4 months. Hence, pembrolizumab was approved by the U.S. FDA for use in patients with PD-L1-positive recurrent or metastatic cervical cancer.

The EMPOWER-Cervical 1/GOG-3016/ENGOT-cx9 trial assessed cemiplimab (PD-1 inhibitor) in metastatic or recurrent patients [35–37]. It significantly improved OS, median OS with cemiplimab was 12 months vs. 8.5 months with chemotherapy. The benefit was seen across patient subgroups, irrespective of PD-L1 status. Cemiplimab also demonstrated higher ORR (16.4% vs. 6.3%) leading to FDA approval [38].

CheckMate 358 is a phase 1/2 trial evaluating nivolumab (anti-PD-1) alone or in combination with ipilimumab (anti-CTLA-4) [39]. In patients treated with ICI combination, the ORR was 31.6% in those with no prior systemic therapy and 23.1% in those with prior treatment. For the combination of lower-dose nivolumab and higher-dose ipilimumab, response rates were higher (45.8% and 36.4% respectively).

There is growing interest in combining ICI with anti-angiogenic agents like bevacizumab. The KEYNOTE-826 trial evaluated pembrolizumab in combination with chemotherapy ± bevacizumab, in metastatic settings (Table 2) [40]. This phase 3 study demonstrated significant improvement in both OS and PFS compared to chemotherapy alone. Adding pembrolizumab led to 36% reduction in the risk of death [hazard ratio (HR) 0.64] and prolonged survival by 12.1 months. Despite these benefits, the treatment was associated with notable AE, including peripheral neuropathy, anaemia, and fatigue. The phase 3 BEATcc trial tested atezolizumab in metastatic or recurrent settings in a biomarker-unselected group, with mandatory use of bevacizumab [41]. Results showed a median PFS of 13.7 months with atezolizumab vs. 10.4 months for standard therapy (HR 0.62, *P* < 0.0001). Median OS was 32.1 months with atezolizumab, compared to 22.8 months (HR 0.68, *P*  =  0.0046).

**Table 2:** Summary of studies highlighting the role of immunotherapy in treatment of recurrent or metastatic cervical cancer

**Author, year**	**Type**	**Sample size**	**Inclusion criteria**	**Treatment**	**Median follow up**	**Disease response**	**Toxicity**
Frenel et al. [33], 2017	Multicentre, phase 1b, single-arm trial	46	Locally advanced, or metastatic PD-L1-positive cervical cancer that had progressed after prior standard therapy	Pembrolizumab every 2 weeks for up to 24 months or until progression, unacceptable toxicity	11 months	17% overall response, median OS 11 months, 1-year PFS 4%	5/24 grade 3 treatment-related AE
Chung et al. [34], 2019	Phase 2	98	Previously treated advanced cervical cancer	Pembrolizumab 200 mg q3 weeks for 2 years or till progression, intolerable toxicity, or physician/patient decision	10.2 months	ORR 12.2%	Grade 3–4 AE 12.2%
Tewari et al. [35], 2021	Randomised phase 3	608	Recurrent cervical cancer who had progressed on platinum-based therapy	Cemiplimab (350 mg every 3 weeks) or investigator’s choice of chemotherapy in 6-week cycles	18.2 months	Median OS 12 months (cemiplimab) vs. 8.5 months	Grade > 3 AE in 45% with cemiplimab vs. 53.4%
Naumann et al. [39], 2019	Phase 1/2	19	Recurrent or metastatic cervical carcinoma	Nivolumab monotherapy (240 mg every 2 weeks for ≤ 2 years) until disease progression, unacceptable toxicity	19.2 months	ORR 26.3%	Any grade AE 12/19
Colombo et al. [40], 2021	Randomised phase 3	584	Persistent, recurrent, or metastatic cervical cancer	Pembrolizumab (200 mg) or placebo every 3 weeks for up to 35 cycles plus platinum-based chemotherapy and, bevacizumab as per investigator discretion	22 months	Median PFS 10.4 months (pembrolizumab) vs. 8.2 months 2-year OS: 53% (pembrolizumab) vs. 41.7%	Grade 3–5 anaemia: 30.3% (pembrolizumab) vs. 26.9% and neutropenia (12.4% vs. 9.7%)

ORR: objective response rate; PD-L1: programmed death-ligand 1; PFS: progression-free survival; AE: adverse events

## Therapeutic vaccines

During pathogenesis, HPV integrates into the host genome, where primarily the oncogenic E6 and E7 genes are retained and expressed, while other HPV genes are lost or remain inactive. This makes neutralizing antibodies from prophylactic vaccines ineffective against these infected cells. To address this, therapeutic HPV vaccines are being developed to clear HPV infections and HPV-associated lesions. These vaccines promote T cell-mediated immunity by targeting early HPV antigens consistently expressed in infected and cancerous cells, with E6 and E7 as primary targets.

**Live vector-based vaccines** utilize bacterial or viral vectors, to stimulate robust cellular and humoral immune responses, often achievable with a single dose. For HPV, *bacterial vectors* like *Listeria monocytogenes* have shown promise by stimulating the activation of both helper and cytotoxic T cells against E6/E7, with the ADXS Listeria-based vaccine currently in phase 2 trials [42]. *Viral vectors*, including adenoviruses and modified vaccinia viruses, can also express HPV antigens in infected cells, engaging the immune system without integrating into the host DNA with some trials showing encouraging immune responses in early-stage cervical and vaginal cancers [43–45]. However, these vaccines must be used with caution, particularly for immunocompromised patients, and pre-existing immunity against the vector can limit efficacy, potentially hindering repeat doses with the same vector type.

**DNA vaccines** are advantageous for HPV immunotherapy due to their safety, stability, and ability to prompt repeated vaccinations without neutralizing antibodies. While DNA vaccines can stimulate both humoral and cellular immunity, they often need adjuvants or delivery enhancements to boost immunogenicity. For example, intramuscular or intradermal injection introduces DNA to host cells, producing antigens that stimulate immune responses. Clinical trials have explored DNA vaccines like pNGVL4a-CRT-E7, which achieved lesion regression in some patients, and GX-188, which showed viral clearance and enhanced T-cell responses in CIN III cases [46, 47]. Electroporation—a DNA delivery technique—has been used to improve transfection efficiency by temporarily permeabilizing cell membranes, promoting antigen uptake and APC recruitment [48]. Other methods, like gene gun and microencapsulation, are also in development to enhance DNA vaccine efficacy [49]. The REVEAL 1 trial (NCT03185013) recently reported that significant HSIL histopathological regression with HPV16/18 virologic clearance at 36 weeks was achieved in 23.7% vs. 11.3% in the placebo group [50]. Another vaccine, INO-3112, targets HPV16/18 E6 and E7 antigens and includes IL-12. Clinical trials have demonstrated its safety and immune response enhancement as an adjunct to chemoradiation in cervical cancer [51].

**Peptide-based vaccines** are stable, safe, and easy to produce, though they often require adjuvants due to low immunogenicity [52]. However, they are MHC-restricted, and HPV peptides must be selected for MHC binding and CTL induction. Short peptides are prone to immune tolerance and limited CD4^+^ activation, while long peptides promote robust CTL responses after processing [53]. In clinical trials, long peptide vaccines targeting HPV16 E6 and E7, paired with adjuvants, enhanced IFN-γ T cell responses in cervical cancer patients. Enhancements like the 4-1BB ligand and bryostatin-I improve DC activation and T cell response [54].

**Protein vaccines** offer a broader MHC response range, allowing APCs to process and present all potential epitopes, making them beneficial for antibody production, though adjuvants are needed to enhance immunogenicity [49]. Clinical trials show promising responses with protein vaccines like SGN-00101 (HPV16 E7-HSP65), which demonstrated clinical response in 78% of CIN III patients, and TA-CIN, which generated HPV16-specific antibodies and T-cell responses in early clinical trials [55, 56]. Combined with the immune modulator imiquimod, TA-CIN showed a 63% remission in patients with VIN II/III, significantly increasing T-cell infiltration in treated lesions [57].

**DCs** are key APCs in immune regulation, activating both innate and adaptive immunity against HPV through antigen presentation via MHC class I and II pathways. DC-based HPV vaccines have shown promise, particularly with HPV16/18 E7 antigens, in inducing targeted T-cell responses in cervical cancer patients [58]. Strategies like incorporating shRNA to silence SOCS1 in DCs enhance vaccine efficacy by supporting Th1 responses, DC activation, and promoting IFN-γ function [59]. Studies using SOCS1-silenced DC vaccines show improved anti-tumor immunity in models, indicating potential for stronger immunotherapeutic effects.

## Adoptive cell therapy

Adoptive T cell therapy (ACT), or T cell-based vaccines, involves extracting a patient’s T cells, expanding or modifying them ex vivo to recognize tumor antigens, and reinfusing them to target tumors. Strategies include are tumor-infiltrating lymphocytes (TILs), TCR-engineered T cells, and chimeric antigen receptor (CAR)-T cells. Efforts to target HPV-specific antigens (E6, E7) have shown limited success in advanced stages though T cells are important for tumor regression [60]. Unlike traditional vaccines, ACTs bypass host APCs, modifying T cells to overcome immune tolerance [61]. Challenges include risks of cytokine release syndrome (CRS), neurotoxicity, and the need for lymphodepletion. Despite these limitations, ACT’s personalized approach holds significant promise for future cancer treatment [62].

**TILs** are T cells derived from tumors, expanded ex vivo, typically in the presence of IL-2 to support selection for tumor antigen reactivity, and then reinfused into the patient. TIL therapy has been explored in cancers such as gastrointestinal, lung, and HPV-associated malignancies. TILs targeting HPV oncogenes E6 and E7 have shown tumor regression in metastatic cervical cancer patients. A preclinical study that isolated and expanded HPV E6-reactive T cells from cervical tumor-draining lymph nodes for potential clinical application. A phase 2 clinical trial demonstrated objective responses in 3/9 patients with HPV-associated cancers using E6/E7-reactive TILs post-lymphodepletion and aldesleukin administration [60]. While the TILs were chosen for their reactivity to HPV-specific antigens, cervical cancer cells may also express additional tumor antigens which could have been a possible target for these expanded T cells. Future research utilizing TILs that are exclusively HPV-specific could clarify whether tumor regression was solely a result of targeting HPV antigens [63]. An alternative to TILs, cytokine-induced killer (CIK) therapy, uses expanded peripheral blood mononuclear cells (PBMCs) rather than tumor-infiltrating immune cells [64]. This approach simplifies tumor targeting while sparing most healthy tissue and has shown efficacy in both solid and hematologic cancers. In cervical cancer, CIK therapy combined with CRT showed short-term effectiveness, supporting further research into its application [65]. Despite this promise, TIL therapy is labour-intensive, with low success rates and limited effectiveness in highly immunosuppressive TME.

**Engineered TCR therapy** expands existing tumor-specific T cells, TCR therapy involves genetically modifying host-derived T cells to express specific TCRs that target tumor antigens unlike TIL therapy. This approach bypasses the immune tolerance typically exhibited by tumors. The cloned, tumor-specific TCR T-cells recognize specific antigen peptides presented by MHC I/II molecules and are reinfused into the patient after expansion [66]. The efficacy of TCR therapy hinges on the successful generation of TCR α and β chains that can effectively bind to tumor targets and their expression in autologous T cells. The identification of tumor cells by engineered TCR cells depends on the abundance of the α/β heterodimer on the cell surface and the receptor’s affinity for the target antigen. Strategies such as modified promoters or mutations in the α and β chains may enhance the effectiveness of engineered TCR T cells [61]. Recent studies suggest that these engineered TCR T cells can recognize E6 and E7-positive tumor cells. A phase 1/2 trial evaluated E6-engineered TCR T cells in HPV16+ cancers, administered alongside lymphocyte depletion and IL-2 [67]. The results indicate that E6 TCR therapy may induce regression in HPV-associated epithelial cancers, warranting further investigation. Multiple ongoing clinical trials are assessing the use of HPV oncoprotein TCR T cells in cervical cancer, with completion expected in the coming years (NCT02858310: E7 TCR T-Cells for HPV-Associated Cancers). Despite these efforts, most successes with TCR therapies have been seen in hematologic malignancies, and the overall therapeutic effects on solid tumors and HPV-associated cancers remain largely unexplored [68].

**CAR-T cell therapy** involves genetically modifying T cells to enhance their specificity for tumor antigens through the introduction of a synthetic recognition structure known as CAR. A key advantage of CAR-T cell therapy is its independence from functional MHC presentation system on cancer cells, which is significant in immunosuppressive TME where MHC expression may be downregulated [69]. Hence, CAR-T cells can potentially eradicate tumors that may not effectively present antigens, unlike TILs, TCR therapies, or traditional immunotherapeutic vaccines. The CAR design consists of an extracellular antigen recognition domain derived from a single-chain variable fragment (scFv) of a mAb, a hinge domain, a transmembrane domain, and an intracellular signalling domain [70]. This structure provides essential co-stimulatory signals necessary for activating effector T cells.

Despite promising results in haematological cancers—such as the FDA-designated “breakthrough” CD19 CAR-T therapy (CTL019), applications in solid tumors, including cervical cancer, are limited due to the highly complex and immunosuppressive TME [71]. Unlike hematologic malignancies, solid tumors present physical barriers such as dense extracellular matrix and irregular vasculature, which hinder CAR-T cell infiltration. Furthermore, cervical cancer often upregulates immunosuppressive molecules like PD-L1 and secretes TGF-β and IL-10, which suppress T-cell activity and proliferation. The TME is enriched with Tregs, myeloid-derived suppressor cells, and tumor-associated macrophages that further dampen the immune response. Additionally, antigen heterogeneity in cervical tumors can lead to immune escape, as tumor cells lacking the targeted antigen can survive and proliferate. These challenges necessitate strategies such as TME-modifying agents, dual-targeting CAR-T cells, or combining CAR-T therapy with ICI to improve efficacy against cervical cancer. Currently, an ongoing Chinese trial is investigating CAR-T cells targeting GD2, PSMA, Muc1, or mesothelin in cervical cancer patients, instead of majority of trials focussing on HPV antigens (NCT03356795).

## Challenges, limitations, and future directions

Despite the promise of immunotherapy in cervical cancer, several challenges hinder its broad adoption. PD-L1 is an imperfect biomarker, necessitating better predictive markers for patient selection [72]. Immune-related AE (irAE), ranging from mild to severe, require vigilant monitoring, with some cases needing treatment discontinuation. Response rates are limited, with some patients showing no benefit, highlighting the need to understand resistance mechanisms. Additionally, the high cost of immunotherapy restricts access, particularly in low-resource settings where cervical cancer incidence is highest, posing significant equity challenges in care delivery [73, 74].

In this era of precision oncology, customized treatments aim to enhance the effectiveness of anticancer drugs by limiting their systemic exposure and focusing on specific molecular markers expressed uniquely by cancer cells. This approach minimizes off-target effects, providing a more targeted and safer therapy and here comes the role of antibody-drug conjugates (ADCs). ADCs combine the precision of a mAb targeting tumor-specific antigens with a cytotoxic chemotherapeutic drug, linked via a chemical bond. The mAb directs the ADC to tumor cells, where it releases its drug payload in the lysosome, leading to targeted cell death through apoptosis or DNA/microtubule disruption [75]. ADCs also exhibit a bystander effect, affecting nearby cells, and modify the TME, bypassing immune suppression mechanisms that often hinder immune-based therapies. ADCs improve target specificity by using antibodies to selectively bind antigens expressed on tumor cells, minimizing off-target effects on healthy tissues. This targeted approach enables the delivery of highly potent cytotoxic drugs directly to cancer cells, overcoming limitations like low therapeutic indices of traditional chemotherapy. Unlike immune-based therapies that rely on an intact immune system, ADCs are less affected by immune evasion strategies, such as PD-L1 overexpression or T-cell exhaustion. By targeting specific tumor antigens and employing cytotoxic payloads, ADCs offer an effective alternative or complement to existing immunotherapies, particularly in cancers with poor immune visibility or resistance to ICI. Tisotumab vedotin (TV) targets tissue factor on cervical cancer cells, using monomethyl auristatin E (MMAE) to cause G2/M arrest and cell death [76, 77].

One primary challenge is achieving sufficient immune cell, antibody, and drug penetration throughout the TME. For instance, CAR-T cells show efficacy in hematologic cancers but face obstacles in solid tumors, where access is often limited. Additionally, “cold tumors”, with low lymphocyte infiltration, are less responsive to ICI. This underscores the importance of research to improve immune cell, antibody, and drug delivery to the TME [78, 79].

Advances in strategies to generate antigen-specific T cells and reverse immunosuppression within the TME offer a pathway to enhanced therapies. Challenges remain, including delivering immune cells and drugs effectively across the TME. HPV is a major etiological factor, making it a clear immunotherapy target. It is important to look for biomarkers beyond PD-L1, focusing on tumor mutational burden, microsatellite instability, and immune signatures to enhance patient selection. Combining immunotherapy with chemotherapy, targeted therapies, or RT may improve response rates and overcome resistance. Personalized immunotherapy, tailored to individual genetic and immune profiles, could optimize efficacy while minimizing toxicity, marking a shift toward more precise and effective treatments.

## Conclusion

Immunotherapy has significantly expanded the treatment landscape for cervical cancer, offering new hope to patients across various stages of the disease. In the neoadjuvant and adjuvant settings, its role remains investigational, while in the concurrent and metastatic/recurrent settings, ICI have already demonstrated clinical benefits, leading to regulatory approvals. As research continues to advance, combining immunotherapy with other therapeutic strategies and identifying predictive biomarkers will be essential to maximizing its potential. Although challenges remain, the promise of immunotherapy in improving the prognosis of cervical cancer patients is becoming increasingly evident.

## Abbreviations

ACT: adoptive T cell therapy
ADCs: antibody-drug conjugates
AE: adverse events
APCs: antigen-presenting cells
CAR: chimeric antigen receptor
CCRT: concurrent chemoradiation
CRT: chemoradiotherapy
CTLA-4: cytotoxic T-lymphocyte-associated protein 4
CTLs: cytotoxic T-cells
DCs: dendritic cells
FDA: Food and Drug Administration
HPV: human papillomavirus
HR: hazard ratio
ICI: immune checkpoint inhibitors
LACC: locally advanced cervical cancer
mAb: monoclonal antibody
MHC: major histocompatibility complex
ORR: objective response rate
PD-1: programmed death-1
PD-L1: programmed death-ligand 1
PFS: progression-free survival
RT: radiotherapy
TCR: T-cell receptor
TILs: tumor-infiltrating lymphocytes
TME: tumor microenvironment
Tregs: regulatory T cells

## Declarations

### Copyright

© The Author(s) 2025.

## References

[1] Bray F, Laversanne M, Sung H, Ferlay J, Siegel RL, Soerjomataram I (2024). Global cancer statistics 2022: GLOBOCAN estimates of incidence and mortality worldwide for 36 cancers in 185 countries. CA Cancer J Clin.

[2] Bhatla N, Aoki D, Sharma DN, Sankaranarayanan R (2021). Cancer of the cervix uteri: 2021 update. Int J Gynaecol Obstet.

[3] Friedlander M, Grogan M, U.S. Preventative Services Task Force (2002). Guidelines for the treatment of recurrent and metastatic cervical cancer. Oncologist.

[4] Yim EK, Park JS (2005). The role of HPV E6 and E7 oncoproteins in HPV-associated cervical carcinogenesis. Cancer Res Treat.

[5] Buchbinder EI, Desai A (2016). CTLA-4 and PD-1 Pathways: Similarities, Differences, and Implications of Their Inhibition. Am J Clin Oncol.

[6] Latchman Y, Wood CR, Chernova T, Chaudhary D, Borde M, Chernova I (2001). PD-L2 is a second ligand for PD-1 and inhibits T cell activation. Nat Immunol.

[7] Ai L, Xu A, Xu J (2020). Roles of PD-1/PD-L1 Pathway: Signaling, Cancer, and Beyond. Adv Exp Med Biol.

[8] Ghosh C, Luong G, Sun Y (2021). A snapshot of the PD-1/PD-L1 pathway. J Cancer.

[9] Weigmann K (2016). Releasing the brakes to fight cancer: The recent discovery of checkpoints has boosted the field of cancer immunotherapy. EMBO Rep.

[10] Njau MN, Kim JH, Chappell CP, Ravindran R, Thomas L, Pulendran B (2012). CD28–B7 Interaction Modulates Short- and Long-Lived Plasma Cell Function. J Immunol.

[11] Driessens G, Kline J, Gajewski TF (2009). Costimulatory and coinhibitory receptors in anti-tumor immunity. Immunol Rev.

[12] Chen FP, Chen K, Huang X, Huang L, Wu HY, Ouyang Y (2024). Neoadjuvant chemo-immunotherapy following concurrent immuno-chemoradiotherapy and immune-maintenance therapy as primary treatment for locally advanced cervical cancer: A prospective, single-arm, phase 2 trial. J Clin Oncol.

[13] Li K, Chen J, Hu Y, Wang YZ, Shen Y, Chen G (2024). Neoadjuvant chemotherapy plus camrelizumab for locally advanced cervical cancer (NACI study): a multicentre, single-arm, phase 2 trial. Lancet Oncol.

[14] Zhu M, Yang M, Zhang J, Yin Y, Fan X, Zhang Y (2021). Immunogenic Cell Death Induction by Ionizing Radiation. Front Immunol.

[15] Krysko DV, Garg AD, Kaczmarek A, Krysko O, Agostinis P, Vandenabeele P (2012). Immunogenic cell death and DAMPs in cancer therapy. Nat Rev Cancer.

[16] Luo H, Ma W, Chen Q, Yang Z, Dai Y (2023). Radiotherapy-activated tumor immune microenvironment: Realizing radiotherapy-immunity combination therapy strategies. Nano Today.

[17] Wan S, Pestka S, Jubin RG, Lyu YL, Tsai YC, Liu LF (2012). Chemotherapeutics and radiation stimulate MHC class I expression through elevated interferon-beta signaling in breast cancer cells. PLoS One.

[18] Lindau D, Gielen P, Kroesen M, Wesseling P, Adema GJ (2013). The immunosuppressive tumour network: myeloid-derived suppressor cells, regulatory T cells and natural killer T cells. Immunology.

[19] Kordbacheh T, Honeychurch J, Blackhall F, Faivre-Finn C, Illidge T (2018). Radiotherapy and anti-PD-1/PD-L1 combinations in lung cancer: building better translational research platforms. Ann Oncol.

[20] Voronova V, Vislobokova A, Mutig K, Samsonov M, Peskov K, Sekacheva M (2022). Combination of immune checkpoint inhibitors with radiation therapy in cancer: A hammer breaking the wall of resistance. Front Oncol.

[21] Cytlak UM, Dyer DP, Honeychurch J, Williams KJ, Travis MA, Illidge TM (2022). Immunomodulation by radiotherapy in tumour control and normal tissue toxicity. Nat Rev Immunol.

[22] Wang X, Zhang H, XinZhang, Liu Y (2024). Abscopal effect: from a rare phenomenon to a new frontier in cancer therapy. Biomark Res.

[23] El-Sayes N, Vito A, Mossman K (2021). Tumor Heterogeneity: A Great Barrier in the Age of Cancer Immunotherapy. Cancers (Basel).

[24] Zhou L, Liu Y, Wu Y, Yang X, Spring Kong FM, Lu Y (2024). Low-dose radiation therapy mobilizes antitumor immunity: New findings and future perspectives. Int J Cancer.

[25] Jiang L, Li X, Zhang J, Li W, Dong F, Chen C (2021). Combined High-Dose LATTICE Radiation Therapy and Immune Checkpoint Blockade for Advanced Bulky Tumors: The Concept and a Case Report. Front Oncol.

[26] Lorusso D, Xiang Y, Hasegawa K, Scambia G, Leiva M, Ramos-Elias P, ENGOT-cx11/GOG-3047/KEYNOTE-A18 investigators (2024). Pembrolizumab or placebo with chemoradiotherapy followed by pembrolizumab or placebo for newly diagnosed, high-risk, locally advanced cervical cancer (ENGOT-cx11/GOG-3047/KEYNOTE-A18): a randomised, double-blind, phase 3 clinical trial. Lancet.

[27] FDA approves pembrolizumab with chemoradiotherapy for FIGO 2014 Stage III-IVA cervical cancer [Internet]. https://www.fda.gov/drugs/resources-information-approved-drugs/fda-approves-pembrolizumab-chemoradiotherapy-figo-2014-stage-iii-iva-cervical-cancer.

[28] Monk BJ, Toita T, Wu X, Vázquez Limón JC, Tarnawski R, Mandai M (2023). Durvalumab versus placebo with chemoradiotherapy for locally advanced cervical cancer (CALLA): a randomised, double-blind, phase 3 trial. Lancet Oncol.

[29] Rodrigues M, Loap P, Dubot C, Durdux C, Bazire L, Minsat M (2024). Combination of nivolumab with chemoradiotherapy for locally advanced cervical cancer: NiCOL phase I trial. J Clin Oncol.

[30] Mayadev JS, Enserro D, Lin YG, Da Silva DM, Lankes HA, Aghajanian C (2020). Sequential Ipilimumab After Chemoradiotherapy in Curative-Intent Treatment of Patients With Node-Positive Cervical Cancer. JAMA Oncol.

[31] Duska LR, Scalici JM, Temkin SM, Schwarz JK, Crane EK, Moxley KM (2020). Results of an early safety analysis of a study of the combination of pembrolizumab and pelvic chemoradiation in locally advanced cervical cancer. Cancer.

[32] Chen J, Shi J, Cao Y, Li C, Li J, Yuan Z (2025). A new treatment approach of toripalimab in combination with concurrent platinum-based chemoradiotherapy for locally advanced cervical cancer: A phase II clinical trial. Int J Cancer.

[33] Frenel JS, Le Tourneau C, O’Neil B, Ott PA, Piha-Paul SA, Gomez-Roca C (2017). Safety and Efficacy of Pembrolizumab in Advanced, Programmed Death Ligand 1-Positive Cervical Cancer: Results From the Phase Ib KEYNOTE-028 Trial. J Clin Oncol.

[34] Chung HC, Ros W, Delord JP, Perets R, Italiano A, Shapira-Frommer R (2019). Efficacy and Safety of Pembrolizumab in Previously Treated Advanced Cervical Cancer: Results From the Phase II KEYNOTE-158 Study. J Clin Oncol.

[35] Tewari KS, Monk BJ, Vergote I, Miller A, de Melo AC, Kim HS (2021). EMPOWER-Cervical 1/GOG-3016/ENGOT-cx9: Interim analysis of phase III trial of cemiplimab vs. investigator’s choice (IC) chemotherapy (chemo) in recurrent/metastatic (R/M) cervical carcinoma. Ann Oncol.

[36] Oaknin A, Monk BJ, Vergote I, Cristina de Melo A, Kim YM, Lisyanskaya AS (2022). EMPOWER CERVICAL-1: Effects of cemiplimab versus chemotherapy on patient-reported quality of life, functioning and symptoms among women with recurrent cervical cancer. Eur J Cancer.

[37] Tewari KS, Monk BJ, Vergote I, Miller A, de Melo AC, Kim HS, Investigators for GOG Protocol 3016 and ENGOT Protocol En-Cx9 (2022). Survival with Cemiplimab in Recurrent Cervical Cancer. N Engl J Med.

[38] Pelosci A Cemiplimab Receives Priority Review From the FDA In Recurrent or Metastatic Cervical Cancer [Internet]. https://www.cancernetwork.com/view/cemiplimab-receives-priority-review-from-the-fda-in-recurrent-of-metastatic-cervical-cancer.

[39] Naumann RW, Hollebecque A, Meyer T, Devlin MJ, Oaknin A, Kerger J (2019). Safety and Efficacy of Nivolumab Monotherapy in Recurrent or Metastatic Cervical, Vaginal, or Vulvar Carcinoma: Results From the Phase I/II CheckMate 358 Trial. J Clin Oncol.

[40] Colombo N, Dubot C, Lorusso D, Caceres MV, Hasegawa K, Shapira-Frommer R, KEYNOTE-826 Investigators (2021). Pembrolizumab for Persistent, Recurrent, or Metastatic Cervical Cancer. N Engl J Med.

[41] Oaknin A, Gladieff L, Martínez-García J, Villacampa G, Takekuma M, De Giorgi U, ENGOT-Cx10–GEICO 68-C–JGOG1084–GOG-3030 Investigators (2024). Atezolizumab plus bevacizumab and chemotherapy for metastatic, persistent, or recurrent cervical cancer (BEATcc): a randomised, open-label, phase 3 trial. Lancet.

[42] Huh WK, Brady WE, Fracasso PM, Dizon DS, Powell MA, Monk BJ (2020). Phase II study of axalimogene filolisbac (ADXS-HPV) for platinum-refractory cervical carcinoma: An NRG oncology/gynecologic oncology group study. Gynecol Oncol.

[43] Borysiewicz LK, Fiander A, Nimako M, Man S, Wilkinson GW, Westmoreland D (1996). A recombinant vaccinia virus encoding human papillomavirus types 16 and 18, E6 and E7 proteins as immunotherapy for cervical cancer. Lancet.

[44] Kaufmann AM, Stern PL, Rankin EM, Sommer H, Nuessler V, Schneider A (2002). Safety and immunogenicity of TA-HPV, a recombinant vaccinia virus expressing modified human papillomavirus (HPV)-16 and HPV-18 *E6* and *E7* genes, in women with progressive cervical cancer. Clin Cancer Res.

[45] Baldwin PJ, van der Burg SH, Boswell CM, Offringa R, Hickling JK, Dobson J (2003). Vaccinia-expressed human papillomavirus 16 and 18 e6 and e7 as a therapeutic vaccination for vulval and vaginal intraepithelial neoplasia. Clin Cancer Res.

[46] Choi YJ, Hur SY, Kim TJ, Hong SR, Lee JK, Cho CH (2020). A Phase II, Prospective, Randomized, Multicenter, Open-Label Study of GX-188E, an HPV DNA Vaccine, in Patients with Cervical Intraepithelial Neoplasia 3. Clin Cancer Res.

[47] Kim TJ, Jin HT, Hur SY, Yang HG, Seo YB, Hong SR (2014). Clearance of persistent HPV infection and cervical lesion by therapeutic DNA vaccine in CIN3 patients. Nat Commun.

[48] Ma B, Maraj B, Tran NP, Knoff J, Chen A, Alvarez RD (2012). Emerging human papillomavirus vaccines. Expert Opin Emerg Drugs.

[49] Lin K, Roosinovich E, Ma B, Hung CF, Wu TC (2010). Therapeutic HPV DNA vaccines. Immunol Res.

[50] INOVIO Announces Positive Results from REVEAL 1, a Phase 3 Pivotal Trial Evaluating VGX-3100, its DNA-based HPV Immunotherapy for the Treatment of High-grade Precancerous Cervical Dysplasia Caused by HPV-16 and/or HPV-18 [Internet]. https://ir.inovio.com/news-releases/news-releases-details/2021/INOVIO-Announces-Positive-Results-from-REVEAL-1-a-Phase-3-Pivotal-Trial-Evaluating-VGX-3100-its-DNA-based-HPV-Immunotherapy-for-the-Treatment-of-High-grade-Precancerous-Cervical-Dysplasia-Caused-by-HPV-16-andor-HPV-18/default.aspx.

[51] Hasan Y, Furtado LV, Tergas AI, Lee NK, Brooks RA, McCall AR (2020). A Phase I trial assessing the safety and tolerability of a therapeutic DNA vaccination against HPV16 and HPV18 E6/E7 oncogenes after chemoradiation for cervical cancer. Int J Radiat Oncol Biol Phys.

[52] Sharma RK, Elpek KG, Yolcu ES, Schabowsky RH, Zhao H, Bandura-Morgan L (2009). Costimulation as a platform for the development of vaccines: a peptide-based vaccine containing a novel form of 4-1BB ligand eradicates established tumors. Cancer Res.

[53] van der Burg SH, Bijker MS, Welters MJP, Offringa R, Melief CJM (2006). Improved peptide vaccine strategies, creating synthetic artificial infections to maximize immune efficacy. Adv Drug Deliv Rev.

[54] Yan W, Chen WC, Liu Z, Huang L (2010). Bryostatin-I: a dendritic cell stimulator for chemokines induction and a promising adjuvant for a peptide based cancer vaccine. Cytokine.

[55] Einstein MH, Kadish AS, Burk RD, Kim MY, Wadler S, Streicher H (2007). Heat shock fusion protein-based immunotherapy for treatment of cervical intraepithelial neoplasia III. Gynecol Oncol.

[56] de Jong A, O’Neill T, Khan AY, Kwappenberg KM, Chisholm SE, Whittle NR (2002). Enhancement of human papillomavirus (HPV) type 16 E6 and E7-specific T-cell immunity in healthy volunteers through vaccination with TA-CIN, an HPV16 L2E7E6 fusion protein vaccine. Vaccine.

[57] Daayana S, Elkord E, Winters U, Pawlita M, Roden R, Stern PL (2010). Phase II trial of imiquimod and HPV therapeutic vaccination in patients with vulval intraepithelial neoplasia. Br J Cancer.

[58] Santin AD, Bellone S, Palmieri M, Zanolini A, Ravaggi A, Siegel ER (2008). Human papillomavirus type 16 and 18 E7-pulsed dendritic cell vaccination of stage IB or IIA cervical cancer patients: a phase I escalating-dose trial. J Virol.

[59] Zhu Y, Zheng Y, Mei L, Liu M, Li S, Xiao H (2013). Enhanced immunotherapeutic effect of modified HPV16 E7-pulsed dendritic cell vaccine by an adeno-shRNA-SOCS1 virus. Int J Oncol.

[60] Stevanović S, Draper LM, Langhan MM, Campbell TE, Kwong ML, Wunderlich JR (2015). Complete regression of metastatic cervical cancer after treatment with human papillomavirus-targeted tumor-infiltrating T cells. J Clin Oncol.

[61] Hossain NM, Chapuis AG, Walter RB (2016). T-Cell Receptor-Engineered Cells for the Treatment of Hematologic Malignancies. Curr Hematol Malig Rep.

[62] Kelderman S, Heemskerk B, Fanchi L, Philips D, Toebes M, Kvistborg P (2016). Antigen-specific TIL therapy for melanoma: A flexible platform for personalized cancer immunotherapy. Eur J Immunol.

[63] Zsiros E, Tsuji T, Odunsi K (2015). Adoptive T-cell therapy is a promising salvage approach for advanced or recurrent metastatic cervical cancer. J Clin Oncol.

[64] Sukari A, Abdallah N, Nagasaka M (2019). Unleash the power of the mighty T cells-basis of adoptive cellular therapy. Crit Rev Oncol Hematol.

[65] Li N, Tian YW, Xu Y, Meng DD, Gao L, Shen W (2019). Combined Treatment with Autologous CIK Cells, Radiotherapy and Chemotherapy in Advanced Cervical Cancer. Pathol Oncol Res.

[66] Draper LM, Kwong ML, Gros A, Stevanović S, Tran E, Kerkar S (2015). Targeting of HPV-16+ Epithelial Cancer Cells by TCR Gene Engineered T Cells Directed against E6. Clin Cancer Res.

[67] Doran SL, Stevanovic S, Adhikary S, Gartner JJ, Jia L, Kwong MLM (2018). Genetically engineered T-cell therapy for HPV-associated epithelial cancers: A first in human, phase I/II clinical trial. J Clin Oncol.

[68] Yang A, Farmer E, Lin J, Wu TC, Hung CF (2017). The current state of therapeutic and T cell-based vaccines against human papillomaviruses. Virus Res.

[69] Dai H, Wang Y, Lu X, Han W (2016). Chimeric Antigen Receptors Modified T-Cells for Cancer Therapy. J Natl Cancer Inst.

[70] Jayaraman J, Mellody MP, Hou AJ, Desai RP, Fung AW, Pham AHT (2020). CAR-T design: Elements and their synergistic function. EBioMedicine.

[71] Zhang H, Ye ZL, Yuan ZG, Luo ZQ, Jin HJ, Qian QJ (2016). New Strategies for the Treatment of Solid Tumors with CAR-T Cells. Int J Biol Sci.

[72] Pasricha S, Durga G, Koyyala VPB, Jajodia A, Gupta G, Mehta A (2024). PD-L1 Testing and Assessment: Practical Considerations for Oncologist and Pathologist. Indian J Med Paediatr Oncol.

[73] Bou Akl I, Berro J, Tfayli A, Shamseddine A, Mukherji D, Temraz S (2020). Current Status and Future Perspectives of Immunotherapy in Middle-Income Countries: A Single-Center Early Experience. World J Oncol.

[74] Patel A, Goldstein DA, Tannock IF (2022). Improving access to immunotherapy in low- and middle-income countries. Ann Oncol.

[75] Fu Z, Li S, Han S, Shi C, Zhang Y (2022). Antibody drug conjugate: the “biological missile” for targeted cancer therapy. Signal Transduct Target Ther.

[76] Breij EC, de Goeij BE, Verploegen S, Schuurhuis DH, Amirkhosravi A, Francis J (2014). An antibody-drug conjugate that targets tissue factor exhibits potent therapeutic activity against a broad range of solid tumors. Cancer Res.

[77] de Bono JS, Concin N, Hong DS, Thistlethwaite FC, Machiels JP, Arkenau HT (2019). Tisotumab vedotin in patients with advanced or metastatic solid tumours (InnovaTV 201): a first-in-human, multicentre, phase 1-2 trial. Lancet Oncol.

[78] Binnewies M, Roberts EW, Kersten K, Chan V, Fearon DF, Merad M (2018). Understanding the tumor immune microenvironment (TIME) for effective therapy. Nat Med.

[79] Li B, Cui Y, Nambiar DK, Sunwoo JB, Li R (2019). The Immune Subtypes and Landscape of Squamous Cell Carcinoma. Clin Cancer Res.

